# Association between reduced cervical extensor muscle mass and postoperative outcomes after single-door laminoplasty in elderly patients with cervical spondylotic myelopathy: a retrospective study

**DOI:** 10.1080/07853890.2026.2653896

**Published:** 2026-05-29

**Authors:** Sun Kai, Wan Haiwu, Zhang Bo, Yin Jinjian, Bo Huang

**Affiliations:** a Department of Orthopedic Surgery, Jiu jiang University Affiliated Hospital, Jiu jiang, China; b Department of Orthopedic Surgery, Jiu jiang Orthopedic Medical Quality Control Center, Jiu jiang, China

**Keywords:** Cervical extensor muscles, cervical spondylotic myelopathy, clinical outcomes, elderly patients, fatty infiltration, open-door laminoplasty

## Abstract

**Background:**

To investigate the association between preoperative cervical extensor muscle fatty infiltration and postoperative clinical and radiographic outcomes after posterior open-door laminoplasty in elderly patients with cervical spondylotic myelopathy (CSM).

**Methods:**

Elderly patients (>60 years) who underwent first-time posterior single-door laminoplasty for multilevel CSM between January 2015 and December 2020 were retrospectively reviewed. All patients had a minimum follow-up of 12 months. Fatty infiltration of cervical extensor muscles was assessed on preoperative MRI using the Goutallier grading system and classified as mild, moderate, or severe. Clinical outcomes (Japanese Orthopaedic Association [JOA] score, Neck Disability Index [NDI], Visual Analog Scale [VAS]), minimal clinically important difference (MCID), perioperative parameters, and cervical sagittal alignment were evaluated.

**Results:**

A total of 101 patients were included (mild: *n* = 45; moderate: *n* = 38; severe: *n* = 18). Baseline characteristics were comparable among groups. Postoperative drainage volume was higher in the moderate (153.4 ± 45.2 mL) and severe groups (145.6 ± 79.5 mL) than in the mild group (99.9 ± 67.4 mL; *p* = 0.001), with longer hospital stay (*p* = 0.042). At 12 months, the severe group showed lower JOA scores (11.95 ± 3.87 vs. 14.69 ± 2.58), reduced JOA recovery rates (55.9%±26.5% vs. 68.3%±23.7%), and lower MCID achievement for JOA and NDI (all *p* < 0.05). Greater loss of cervical lordosis was observed in the severe group (C2–C7 Cobb angle: 3.57 ± 9.62° vs. 7.34 ± 9.80°; *p* < 0.001).

**Conclusion:**

Severe fatty infiltration of cervical extensor muscles is associated with poorer neurological recovery, prolonged hospitalization, and sagittal imbalance after laminoplasty in elderly CSM patients. Preoperative MRI-based muscle quality assessment may improve risk stratification and guide surgical planning and postoperative rehabilitation.

## Introduction

Cervical spondylotic myelopathy (CSM) is the most common cause of spinal cord dysfunction in adults worldwide. It is caused by the progressive degenerative changes in the cervical spine that lead to spinal cord compression. CSM primarily affects individuals over the age of 50, with a higher prevalence in males. Its incidence is increasing as the global population ages [1]. Studies have shown that up to 60% of people over the age of 50 show radiological evidence of cervical spondylosis, and 10–15% develop cervical spondylotic myelopathy [2]. Risk factors include genetic susceptibility, repetitive neck movements, and a history of trauma. CSM poses a significant challenge to healthcare, requiring long-term management and often necessitating surgical intervention. In developed countries, spinal disorders, including CSM, contribute to economic costs exceeding billions of dollars annually. Generally, for patients with multilevel involvement and a positive K-line, posterior cervical open-door laminoplasty is the preferred surgical approach [3–5]. However, the factors influencing postoperative outcomes are numerous. In elderly patients, it is crucial not only to achieve favorable surgical outcomes but also to ensure surgical safety, minimize adverse effects, and accelerate recovery [6,7]^.^ Recent studies suggest that sarcopenia may be an independent and significant risk factor for predicting adverse events in elderly spinal surgery patients [8,9]. Sarcopenia is characterized by a reduction in skeletal muscle mass, strength, and endurance. Previous expert consensus on sarcopenia identified low muscle mass, diminished muscle strength, and impaired physical function as key components [10–12]. However, for many spine patients, pain or nerve impairment may prevent comprehensive assessments of muscle strength and other related factors [13,14]^.^ Currently, there is no consensus in spinal surgery regarding the most appropriate method to diagnose sarcopenia or to determine clinically relevant thresholds [15]. Most studies describe muscle mass through advanced imaging of muscle area or fat infiltration *via* CT or MRI. In spinal surgery, as in other surgical fields, advanced imaging assessment remains the most common method for evaluating muscle mass [16–19]. An increasing number of studies are using muscle quality as a key indicator for assessing surgical outcomes in elderly individuals undergoing spinal procedures, particularly in studies of lumbar spine surgery where parameters such as the cross-sectional area of the multifidus muscle, the degree of multifidus muscle fat infiltration, and the paraspinal lumbar vertebral index (PLVI) have been investigated as indicators of central sarcopenia and predictors of surgical tolerance and outcomes in elderly patients [20–23]^.^ However, there is limited reporting on the impact of decreased cervical paraspinal extensor muscle mass on postoperative outcomes in elderly patients undergoing cervical spine surgery. This study aims to retrospectively analyze a cohort of elderly patients, who were classified on the basis of the severity of paraspinal muscle fat infiltration observed on preoperative cervical MR images. The goal of this study was to evaluate the association between cervical extensor muscle fat infiltration on the outcomes of laminoplasty. Our objective is to assess whether this factor serves as a potential prognostic indicator in elderly patients undergoing posterior cervical laminoplasty.

## Methods

After obtaining approval from the Institutional Review Board, we retrospectively reviewed 116 elderly patients (aged 60–82 years) who underwent posterior cervical single-door laminoplasty between January 2015 and December 2020. The inclusion criteria for this study were: 1) aged over 60 years; 2) undergoing first-time cervical surgery for multilevel cervical spinal canal stenosis (≥3 levels); 3) with clinical presentations of cervical spondylotic myelopathy (CSM); and 4) availability of complete preoperative clinical and imaging data, along with a minimum follow-up of 12 months postoperatively, including reassessment of clinical scores and cervical radiographs.The exclusion criteria included patients with: 1) acute spinal cord injury; 2) ossification of the posterior longitudinal ligament (OPLL); 3) history of previous cervical spine surgery or requiring reoperation for any reason during the study period.

### Surgical technique

All surgeries were performed by a professionally trained spine surgeon with at least 10 years of experience. Patients were placed under general anesthesia with endotracheal intubation and positioned prone on a specialized plaster bed to maintain cervical extension in a neutral position. The surgical field was disinfected *via* standard protocols, and sterile drapes were applied. A standard posterior midline cervical approach was used, with careful layer-by-layer dissection [24]. Muscle attachments to C2 and C7 were preserved while the laminae and facet joints from C3 to C7 were exposed, followed by removal of the upper third of the relevant spinous processes. On the basis of preoperative imaging, additional procedures were performed as needed: a C3 laminectomy, dome laminectomies of C2 and/or C7, or foraminotomy. For C4–C6 laminoplasty, dome resections of C3 and C7 were completed; for C3–C6 laminoplasty, dome resections of C2 and C7 were performed. The symptomatic side was designated the ‘open-door’ side, whereas the opposite side served as the ‘hinge-door’ side. Bite forceps were used to groove both sides along the medial edge of the facet joints, approximately 2.5 mm from the midline. On the hinge side, only the outer cortical bone was removed, whereas on the open-door side, both the inner and outer cortical layers were excised. The lamina was gently elevated toward the hinge side, and a nerve dissector was used to carefully release any adhesions. The ligamentum flavum was resected with rongeurs to fully open the lamina, and the superior and inferior ends of the opened laminae and ligamentum flavum were transected for complete decompression, which was confirmed by observing clear posterior displacement and pulsation of the dural sac. Appropriate-sized Z-shaped titanium plates were selected on the basis of the degree of opening and were used to secure the elevated lamina to the corresponding lateral mass on the open side. Each plate was fixed with four screws. After hemostasis was ensured and the wound was irrigated, a negative pressure drain was placed, and the wound was closed in layers. Postoperatively, drainage was monitored daily, with the drain being removed once the output was less than 50 ml within 24 h. All patients were fitted with a soft cervical collar for three weeks postoperatively.

### Imaging assessment

**Figure 1.**
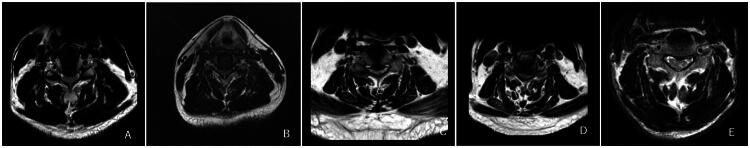
T2 axial images obtained at the C5/6 were used for fatty infiltration grading. A Goutalier 0; B Goutalier 1; C Goutalier 2; D Goutalier 3; E Goutalier 4.

**Figure 2.**
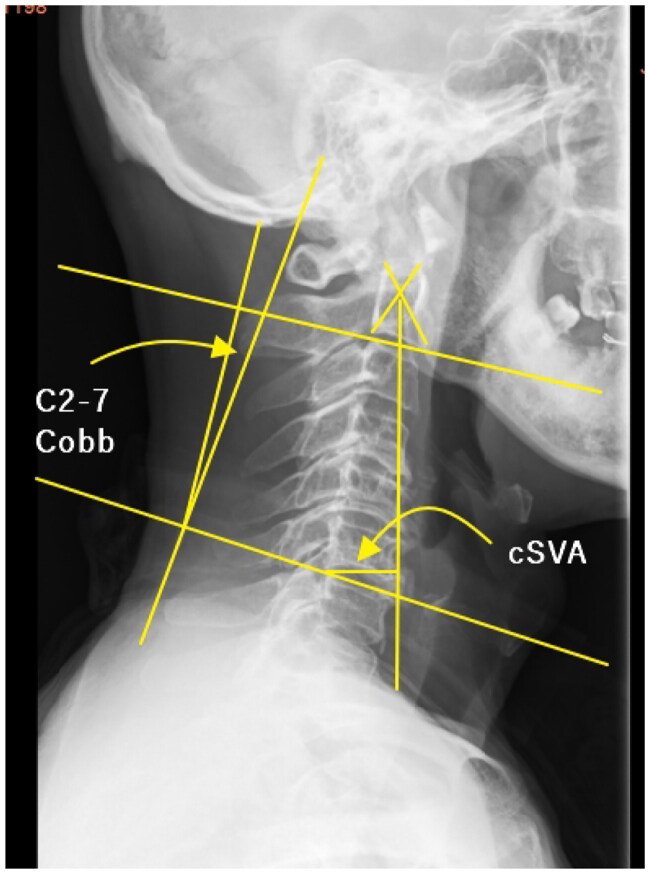
Cobb angle; (2) Sagittal vertical axis (SVA): radiological evaluation of the cervical sagittal alignment parameters. (1) C2–C7.

**Figure 3.**
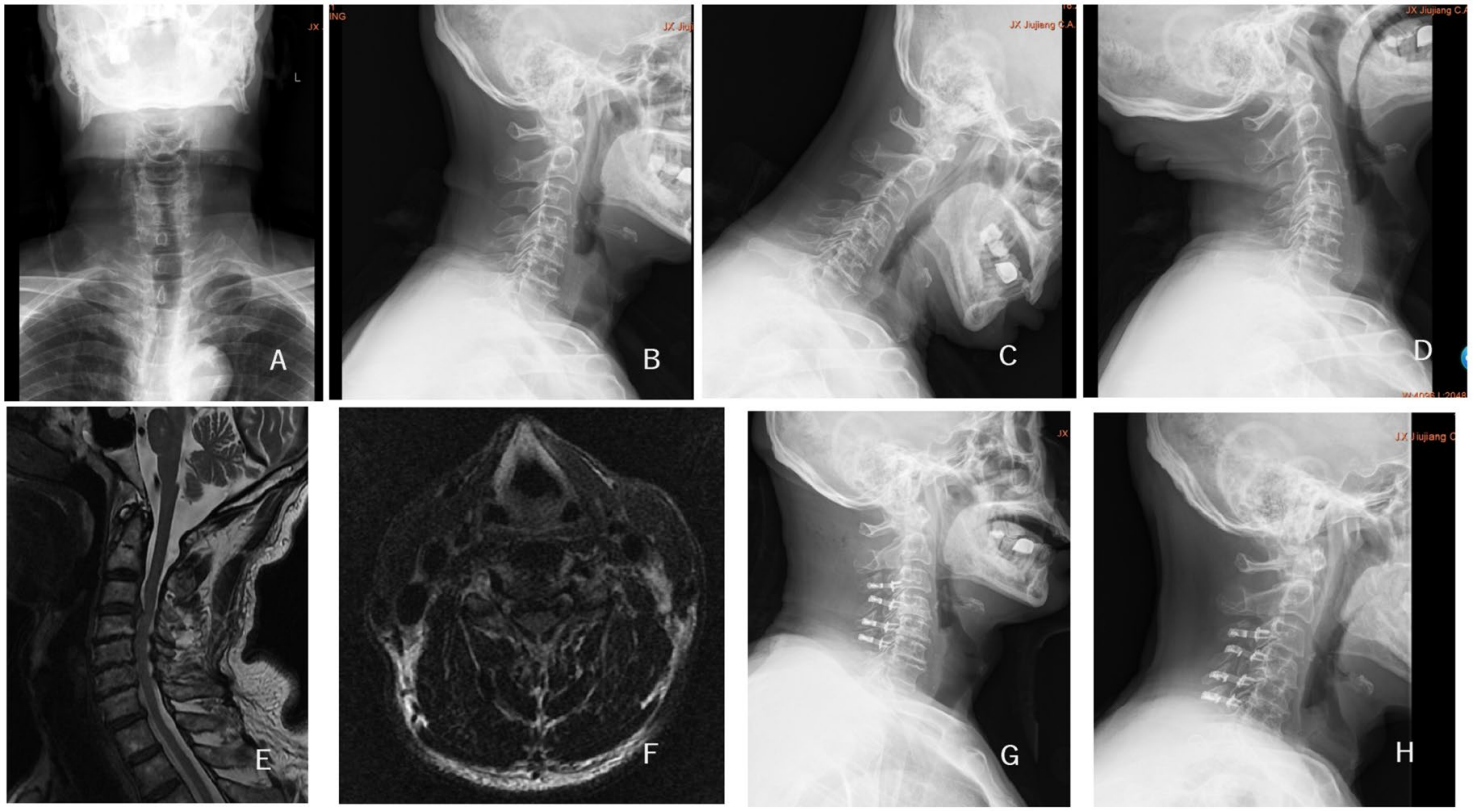
75-year-old male patient was diagnosed with multilevel cervical spondylotic myelopathy, with cervical extensor muscle fatty infiltration classified as Goutalier grade 1. A, B: Preoperative anteroposterior and lateral cervical DR; C, D: Preoperative cervical hyperextension and hyperflexion DR; E, F: Preoperative cervical MR; G: Postoperative lateral cervical DR at 1 week; H: Follow-up lateral cervical DR at 12 months, founding that the cervical lordosis showed little change compared to preoperative.

**Figure 4.**
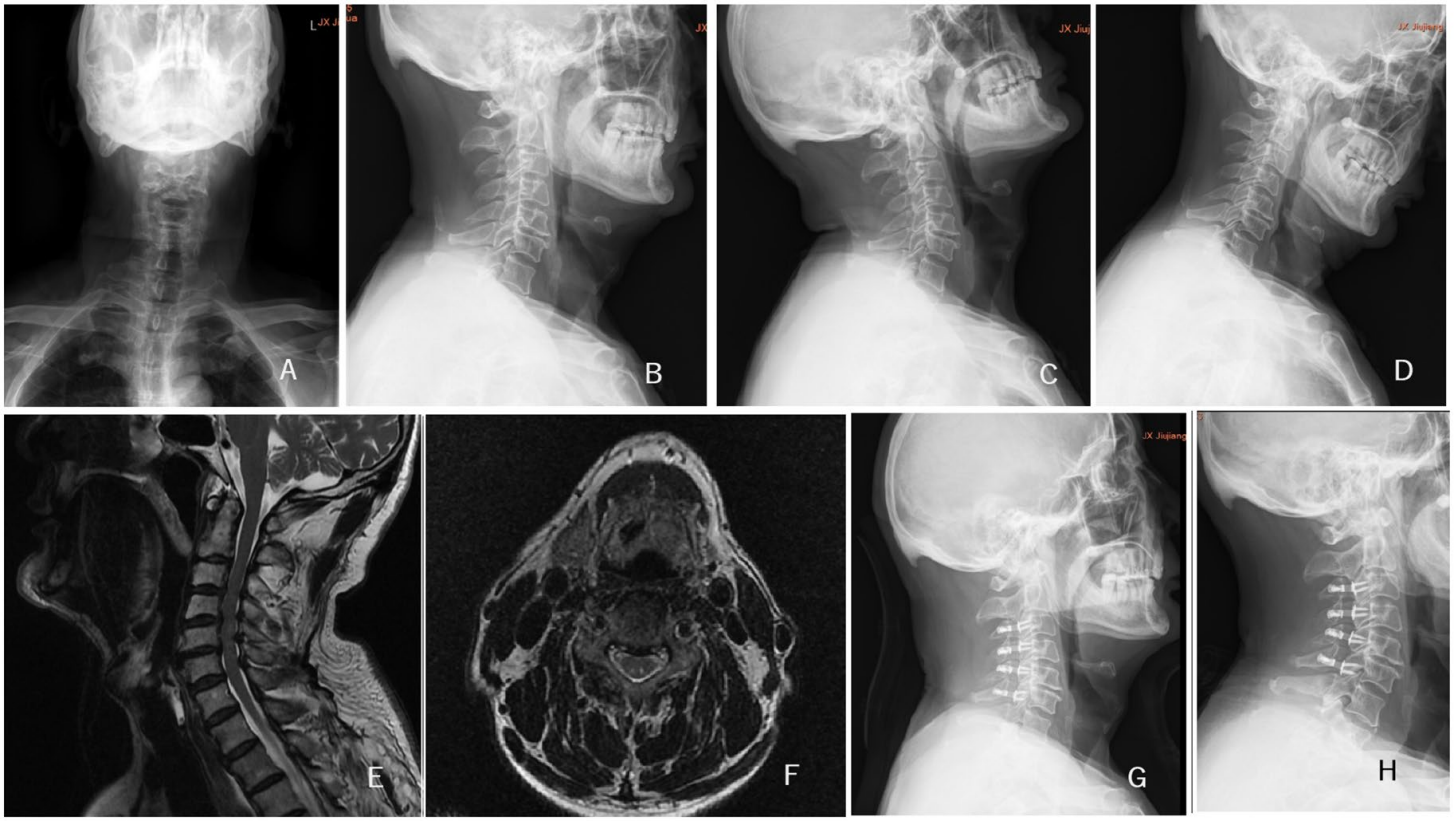
A 72-year-old female patient was diagnosed with multilevel cervical spondylotic myelopathy, with cervical extensor muscle fatty infiltration classified as Goutalier grade 2. A, B: Preoperative anteroposterior and lateral cervical DR; C, D: Preoperative cervical hyperextension and hyperflexion DR; E, F: Preoperative cervical MR; G: Postoperative lateral cervical DR at 1 week; H: Follow-up lateral cervical DR at 12 months, founding that the maintenance of cervical lordosis is relatively good.

**Figure 5.**
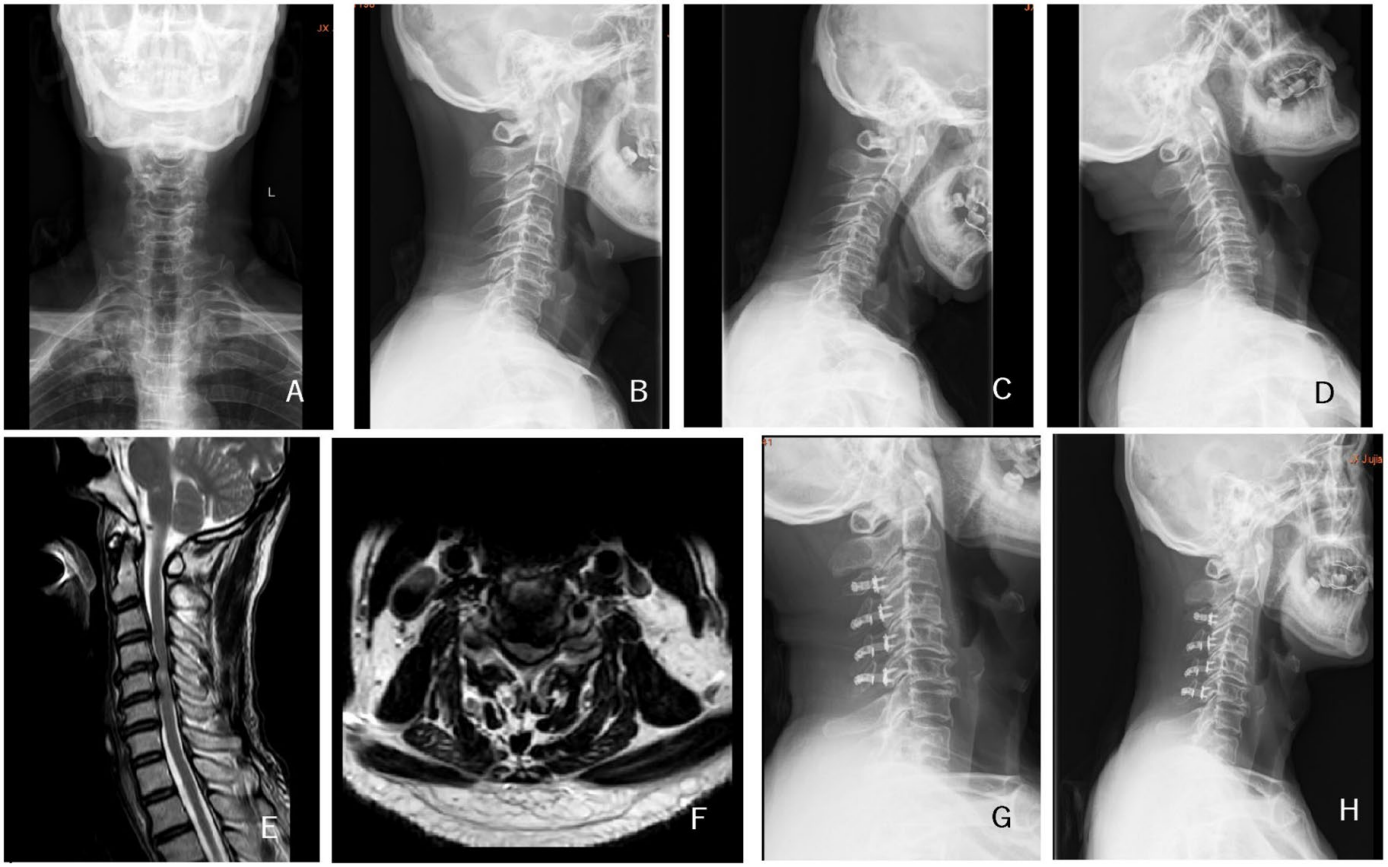
A 68-year-old female patient was diagnosed with multilevel cervical spondylotic myelopathy, with cervical extensor muscle fatty infiltration classified as Goutalier grade 3. A, B: Preoperative anteroposterior and lateral cervical DR; C, D: Preoperative cervical hyperextension and hyperflexion DR; E, F: Preoperative cervical MR; G: Postoperative lateral cervical DR at 3 days; H: Follow-up lateral cervical DR at 12 months, revealing the development of local kyphotic deformity in the cervical spine.

All measurements were conducted by professionally trained radiologists. Each patient underwent MRI according to a standardized protocol, with measurements taken at the C5 lower endplate level. Using the hospital’s picture archiving and communication system (PACS), manual delineation of the paraspinal extensor muscles and surrounding fat was performed on T2-weighted axial images to define the regions of interest (ROIs). ImageJ software (U.S. National Institutes of Health, Bethesda, MD, USA) was used to process the MR images and classify fatty infiltration on the basis of the Goutalier grading system [25]. A grade of 0 indicates no visible fat streaks in the muscle, a grade of 1 represents minimal fatty streaks, a grade of 2 represents more muscle than fat, a grade of 3 represents equal amounts of muscle and fat, and a grade of 4 reflects more fat than muscle (Figure 1). Patients were further stratified into three categories on the basis of a modified Fuchs system: mild (Goutalier 0–1), moderate (Goutalier 2), and severe (Goutalier 3–4) [26]^.^ Preoperative X-rays, including lateral and anteroposterior views, were obtained to assess cervical alignment, particularly focusing on sagittal balance and cervical lordosis (Figures 2–5). All measurements were conducted by two professionally trained radiologists who were blinded to the clinical group assignments and postoperative outcome data of the patients at the time of image analysis.

### Statistical analysis

A pre-study power analysis was performed with G*Power 3.1. Using an ANOVA model for repeated measures (within-between interaction), with α = 0.05, power = 0.90, an estimated within-group correlation of 0.48, and an effect size of 0.25, the analysis determined that a minimum of 16 patients per group was required. Statistical analyses were performed *via* SPSS version 23.0 (IBM Corporation, Armonk, NY, USA). The Shapiro–Wilk test was used to assess data normality. Continuous variables with normal distribution are presented as mean ± standard deviation, while non-normally distributed data are presented as median (range). Categorical variables are expressed as frequencies and percentages. For comparisons across the three patient groups (mild, moderate, severe fat infiltration) at baseline, one-way ANOVA or the Kruskal–Wallis *H* test was used for continuous variables, and the chi-square test or Fisher’s exact test was used for categorical variables. To analyze the changes in clinical scores (e.g. NDI, VAS, JOA) and radiographic parameters (Cobb angle, cSVA) over time (preoperative vs. 12-month postoperative) between the three groups, a two-way repeated-measures ANOVA was employed, with “patient group” as the between-subjects factor and “time” as the within-subjects factor. This allowed us to test for main effects of group and time, as well as their interaction (group × time). A *p* value of less than 0.05 was considered statistically significant.

## Results

A total of 101 patients had complete follow-up data at the final evaluation. There were no statistically significant differences in the general clinical characteristics among the three patient groups (Table 1). Regarding surgery-related parameters, the number of operated segments, operative time, and intraoperative blood loss showed no significant differences. However, postoperative drainage volume was significantly higher in the moderate (153.41 ± 45.20 mL) and severe (145.56 ± 79.49 mL) fat infiltration groups compared to the mild group (99.91 ± 67.39 mL; *p* = 0.001). The length of hospital stay was also significantly longer in the moderate (11.73 ± 3.94 days) and severe (13.65 ± 4.21 days) groups than in the mild group (7.45 ± 2.42 days; *p* = 0.042, Table 2). Preoperative clinical scores, including the Neck Disability Index (NDI), Japanese Orthopaedic Association (JOA) score, and Visual Analog Scale (VAS) scores for neck and arm pain, were comparable across all three groups (all *p* > 0.05). At the 12-month follow-up, significant intergroup differences emerged in several key outcomes. The JOA score improved to 14.69 ± 2.58 in the mild group, which was significantly higher than in the moderate (12.89 ± 2.44) and severe (11.95 ± 3.87) groups (*p* = 0.001). Accordingly, the JOA recovery rate was highest in the mild group (68.28 ± 23.71%), followed by the moderate (60.69 ± 22.94%) and severe (55.86 ± 26.54%) groups (*p* = 0.032). Similarly, postoperative NDI and neck VAS scores were significantly lower (indicating better outcomes) in the mild group (7.56 ± 10.45 and 1.22 ± 1.34, respectively) compared to the moderate and severe groups (e.g. neck VAS: 2.27 ± 1.66 and 2.72 ± 1.81; *p* = 0.017, Table 3). The proportion of patients achieving the minimal clinically important difference (MCID) for NDI, neck VAS, and JOA scores was also significantly higher in the mild group (e.g. JOA MCID: 35/45) than in the moderate (24/38) and severe (9/18) groups (*p* = 0.012). Radiographic analysis revealed that while preoperative cervical sagittal alignment parameters (C2–C7 Cobb angle and cervical sagittal vertical axis, cSVA) were similar, significant differences developed by the 12-month follow-up. The C2–C7 Cobb angle, indicating lordosis, was better maintained in the mild group (7.34 ± 9.80°) than in the moderate (4.27 ± 7.59°) and severe (3.57 ± 9.62°) groups (*p* < 0.001). Concurrently, the cSVA, reflecting sagittal balance, was smaller (indicating better balance) in the mild group (21.36 ± 12.29 mm) compared to the moderate (26.10 ± 9.71 mm) and severe (26.52 ± 12.65 mm) groups (*p* = 0.017, Table 4).

**Table 1.** Comparisons of baseline data.

	Mild (*n*=45)	Moderate (*n*=38)	Severe (*n*=18)	All patients (*n*=101)	*P* value
Age at surgery (years)	75.54 ± 10.22	74.86 ± 7.39	76.71 ± 8.87	75.95 ± 8.72	0.099
Gender (male/female)	25(M)/20(F)	22(M)/16(F)	10(M)/8(F)	55(M)/47(F)	0.278
BMI	26.2 ± 8.4	25.7 ± 7.6	26.6 ± 6.9	26.2 ± 9.8	0.856
Diabetes	9/45	7/38	4/18	20/101	0.072
Active tobacco use	14/45	12/38	8/18	34/101	0.567
Symptom persistence period (M)	17.6 ± 5.9	15.2 ± 8.4	18.0 ± 7.9	16.7 ± 7.0	0.632
MRI T2 hyperintensity area(Y/N)	16/29	15/23	7/11	38/63	0.329

**Table 2.** Comparisons of surgery-related parameter.

	Mild (*n*=45)	Moderate (*n*=38)	Severe (*n*=18)	All patients (*n*=101)	*P* value
Number of surgical segments	31(3**–**6)	26(3**–**6)	10(3–6)	67/34 (3–6)/(4–6)	0.97
	14(4–6)	12(4–6)	8(4–6)		
Operation time (min)	132.53 ± 40.32	123.92 ± 51.47	136.67 ± 47.93	130.97 ± 58.799	0.120
Amount of bleeding(ml)	121.44 ± 80.54	137.91 ± 71.32	131.53 ± 82.20	132.56 ± 47.92	0.117
Postoperative drainage volume(ml)	99.91 ± 67.39	153.41 ± 45.20	145.56 ± 79.49	145.67 ± 65.40	0.001
Hospital stays (d)	7.45 ± 2.42	11.73 ± 3.94	13.65 ± 4.21	10.89 ± 4.58	0.042

**Table 3.** Comparisons of surgical outcomes.

	Mild (*n*=45)	Moderate (*n*=38)	Severe (*n*=18)	All patients (*n*=101)
**NDI score**				
Pre-operation	21.52 ± 8.32	23.45 ± 7.96	22.72 ± 9.10	0.261
Postoperative 12 months	7.56 ± 10.45	10.88 ± 9.38	12.20 ± 7.49	<0.001
MCID	36/45	25/38	10/18	0.018
**VAS neck**				
Pre-operation	5.23 ± 1.54	5.70 ± 1.80	5.39 ± 1.29	0.092
Postoperative 12 months	1.22 ± 1.34	2.27 ± 1.66	2.72 ± 1.81	0.017
MCID	34/45	24/38	9/18	0.023
**VAS arm**				
Pre-operation	4.57 ± 1.35	4.89 ± 1.33	5.10 ± 1.44	0.126
Postoperative 12 months	0.53 ± 1.72	0.79 ± 1.36	0.64 ± 1.86	0.591
MCID	33/45	28/38	12/18	0.063
**JOA scores**				
Pre-operation	9.56 ± 1.75	10.29 ± 2.37	9.42 ± 2.93	0.946
Postoperative 12 months	14.69 ± 2.58	12.89 ± 2.44	11.95 ± 3.87	0.001
JOA recovery rate (%)	68.28 ± 23.71	60.69 ± 22.94	55.86 ± 26.54	0.032
MCID	35/45	24/38	9/18	0.012

**Table 4.** Comparisons of cervical radiographic alignment parameters.

	Mild (*n*=45)	Moderate (*n*=38)	Severe (*n*=18)	All patients (*n*=101)
cSVA				
Preoperative	18.58 ± 10.32	19.93 ± 11.55	20.29 ± 10.46	0.196
First time Postoperative	25.45 ± 12.42	26.60 ± 13.68	27.24 ± 12.58	0.319
Postoperative 12 months	21.36 ± 12.29	26.10 ± 9.71	26.52 ± 12.65	0.017
C2–C7 Cobb angle				
Preoperative	12.18 ± 9.26	13.9 ± 10.53	12.92 ± 8.39	0.185
First time postoperative	8.54 ± 8.51	7.52 ± 8.74	7.70 ± 7.42	0.313
Postoperative 12 months	7.34 ± 9.80	4.27 ± 7.59	3.57 ± 9.62	<0.001

## Discussion

This study used MRI to measure the area of cervical extensor muscles and the degree of fat infiltration to assess muscle quality. The findings demonstrate that cervical extensor muscle quality may predict adverse surgical outcomes in elderly patients undergoing single-door laminoplasty. To the best of our knowledge, this is the first report of such a finding. Open-door laminoplasty is currently the most commonly employed surgical technique for elderly patients with multilevel cervical spondylotic myelopathy (CSM), with generally reliable postoperative results [27]^.^ Previous studies have shown that cervical muscles are essential for maintaining overall spinal alignment, mobility, and stability [28]. However, there is limited research on how muscle quality specifically affects the outcomes of cervical laminoplasty. One known study assessed sarcopenia using dual-energy X-ray absorptiometry (DEXA) to measure the skeletal muscle index (SMI) of the limbs. The study found that patients with sarcopenia had greater postoperative C2–C7 sagittal vertical axis (SVA) values compared to non-sarcopenic patients, with correspondingly poorer surgical outcomes. This suggests that preoperative assessment of sarcopenia may be crucial for predicting the success of cervical decompression surgeries [29]. However, the DEXA scan used in that study is not a routine preoperative test, and conducting it for every patient undergoing laminoplasty would significantly increase healthcare costs .In contrast, MRI-based measurement of cervical extensor muscle quality, which is part of the standard preoperative assessment, does not add any additional cost. Another study evaluated a dataset of 757 patients with degenerative cervical myelopathy (DCM) to determine the impact of frailty on postoperative neurological outcomes. The study found that increased levels of frailty in patients undergoing DCM surgery were associated with a decline in baseline function and quality of life measures. This research highlights the potential modifiable factors in frail DCM patients that should be optimized preoperatively to improve functional outcomes. However, the study’s frailty scale assessment was highly subjective, and it lacked objective radiological evidence [30]^.^ According to the results of this study, preoperative measurement of fat infiltration in cervical extensor muscles can serve as an accurate predictor of surgical outcomes, offering a convenient and cost-effective method for clinical evaluation.

One of the key findings of this study is the significant difference in postoperative drainage and hospital stay among the three patient groups. Patients with moderate-to-severe fat infiltration had significantly higher drainage volumes compared to those with mild fat infiltration, while the mild infiltration group had a notably shorter hospital stay. This possibly indicates that reduced cervical extensor muscle quality has a negative impact on perioperative recovery in elderly patients, with better muscle quality correlating with faster recovery. Though studies on the relationship between cervical muscle quality and hospital stay are uncommon, similar findings have been widely reported in lumbar spine research. A study evaluating perioperative risks in thoracolumbar surgery based on multifidus muscle fat infiltration at the L4 vertebral level found that sarcopenic patients had a 1.7-fold longer hospital stay. The study concluded that multifidus fat infiltration could be an effective tool for assessing perioperative risk and sarcopenia [31]. Another study examining multifidus fat infiltration found that severe fat infiltration resulted in increased postoperative drainage and a higher risk of infection after lumbar fusion surgery, with severe fat infiltration being an independent risk factor for postoperative infection [32]^.^ Several large surgical studies have explored the association between muscle fat infiltration and prolonged hospital stays [33–36], producing results similar to ours. Higher levels of fat infiltration are linked to extended hospitalization due to complications related to muscle quality deterioration and sarcopenia. Sarcopenia also leads to reduced physical function and resilience to surgical stress, causing a heightened postoperative inflammatory response and longer hospital stays [37]. A study on patient satisfaction after cervical laminoplasty found that short-term dissatisfaction was primarily related to costs and hospital stays, while long-term satisfaction was based on functional recovery and axial pain [38]. Thus, we suggest preoperative assessment of muscle quality, particularly the degree of fat infiltration, to predict hospital stay duration. Accurate predictions can help align patient expectations, improve trust, and enhance short-term satisfaction.

Posterior spinal muscles play a critical role in maintaining sagittal balance. One study demonstrated a significant relationship between paraspinal muscle volume and cervical alignment parameters as well as disc degeneration [39]. Specifically, the deep extensor muscles, such as the semispinalis cervicis and multifidus, act as dynamic stabilizers. They generate a posterior extension moment that counteracts the forward gravitational pull of the head. Severe fatty infiltration and atrophy within these muscles diminish their cross-sectional area and contractile efficiency, thereby reducing this crucial counterbalancing moment. Another study used MRI to measure the cross-sectional area (CSA) of cervical muscles in order to investigate the biomechanics of reduced cervical lordosis and the relationship between cervical lordosis angle and muscle condition. It found a significant association between cervical muscle imbalance, particularly extensor muscle weakness, and the loss of cervical lordosis [40]^.^ For patients with multilevel cervical spondylotic myelopathy (CSM) who were scheduled for surgery, measurements of the deep extensor muscles, including the multifidus and semispinalis capitis, revealed that these muscles play a crucial role in maintaining cervical sagittal balance. The area of these deep extensor muscles was correlated with the severity of neck symptoms, underscoring the importance of cervical extensor muscles in postoperative outcomes [41]. This study aligns with our findings, though it did not account for muscle quality based solely on muscle area, which could introduce individual variability and potential errors in the results. Our study confirms that MRI-based quantitative assessment of muscle area and fat infiltration ratio provides a more reliable evaluation of muscle quality. The results indicate that this method is essential for maintaining sagittal balance following open-door laminoplasty. Following laminoplasty, the posterior tension band is altered. In patients with robust extensors, the muscles may compensate and help maintain alignment. However, in the context of severe sarcopenia, the weakened muscles fail to provide adequate dynamic support, potentially leading to progressive kyphotic change under the continuous anterior gravitational force. Similar studies have reported comparable findings in cervical deformity surgeries. One study on posterior cervical kyphosis surgery found that fat infiltration and atrophy of the extensor muscle group were associated with worsened postoperative outcomes, serving as a key predictor of sagittal alignment [42]. Another retrospective study on posterior cervical surgeries found that the C2–C7 extensor muscle fat infiltration ratio was the strongest predictor of postoperative cervical sagittal vertical axis (cSVA) after one year, demonstrating the significance of cervical extensor muscle quality for sagittal stability [43]^.^ Given the well-established link between sagittal imbalance and poor surgical outcomes, we hypothesize that sarcopenic patients undergoing laminoplasty lack sufficient extensor muscle strength to counteract the loss of lordosis and the progression of sagittal imbalance. This results in persistent neck disability and pain, contributing to suboptimal outcomes. Recent literature has reported several modifications to posterior cervical approaches. One notable improvement is the “muscle-sparing” technique in open-door laminoplasty. A comparative study examined the postoperative pain and outcomes of different laminoplasty techniques, including unilateral laminoplasty (UL), double-door laminoplasty (DL), and roof-lifting laminoplasty (RL), in patients with multilevel degenerative cervical myelopathy (MDCM) [44]. The findings revealed that the three muscle-preserving laminoplasty techniques exhibited similar yet superior short-term surgical outcomes compared to LP. This study indirectly underscores the critical significance of posterior extensor muscles in posterior cervical surgical approaches.

This study has several limitations. First, as a single-center retrospective analysis with a limited sample size and a follow-up period of 12 months, the generalizability of our results may be constrained, and potential biases inherent to the observational design cannot be ruled out. Second, the retrospective nature of the study precludes the establishment of causality; while cervical extensor muscle fatty infiltration was significantly associated with poorer postoperative outcomes, this relationship warrants validation through prospective or interventional studies. Third, assessments of symptom relief may be subject to patient-reported subjectivity, and variations in individual responses to medication or rehabilitation were not controlled for, which could introduce confounding. Fourth, technical variations in MRI acquisition (e.g. slice angles and levels) and individual anatomical differences may affect the precision of muscle boundary delineation and fat infiltration quantification, despite efforts to standardize measurements. Additionally, radiographic analysis was limited to specific parameters (C2–C7 Cobb angle and cSVA), and other potential contributing factors were not comprehensively evaluated. Finally, this study assessed only preoperative muscle quality; postoperative changes in cervical extensor muscles were not examined, highlighting the need for future longitudinal imaging studies to evaluate dynamic morphological and compositional adaptations following surgery.

## Conclusion

Preoperative assessment of the fat infiltration ratio in the cervical extensor muscles through MRI may predict adverse surgical outcomes in elderly patients undergoing single-door laminoplasty. Based on these findings, MRI-based evaluation of cervical extensor muscle quality should be incorporated into preoperative risk stratification to guide surgical planning and postoperative rehabilitation in elderly CSM patients. Spine surgeons should assess the condition of cervical extensor muscles when planning laminoplasty for elderly patients with multilevel cervical disease.

## Data Availability

The data that support the findings of this study are not openly available due to reasons of sensitivity and are available from the corresponding author upon reasonable request.

## References

[1] Bakhsheshian J, Mehta VA, Liu JC. Current diagnosis and management of cervical spondylotic myelopathy. Global Spine J. 2017;7(6):572–586. doi: 10.1177/2192568217699208. 28894688 PMC5582708

[2] McCormick JR, Sama AJ, Schiller NC, et al. Cervical spondylotic myelopathy: a guide to diagnosis and management. J Am Board Fam Med. 2020;33(2):303–313. doi: 10.3122/jabfm.2020.02.190195. 32179614

[3] Boogaarts HD, Bartels RH. Prevalence of cervical spondylotic myelopathy. Eur Spine J. 2015;24 Suppl 2:139–141. doi: 10.1007/s00586-013-2781-x. 23616201

[4] Fehlings MG, Tetreault LA, Wilson JR, et al. Cervical spondylotic myelopathy: current state of the art and future directions. Spine (Phila Pa 1976). 2013;38(22 Suppl 1):S1–S8. doi: 10.1097/BRS.0b013e3182a7e9e0. 23962994

[5] Rao RD, Gourab K, David KS. Operative treatment of cervical spondylotic myelopathy. J Bone Joint Surg Am. 2006;88(7):1619–1640. doi: 10.2106/JBJS.F.00014. 16818991

[6] Machino M, Ando K, Kobayashi K, et al. Risk factors for poor outcome of cervical laminoplasty: multivariate analysis in 505 patients with cervical spondylotic myelopathy. Spine (Phila Pa 1976). 2021;46(5):329–336. doi: 10.1097/BRS.0000000000003783. 33156275

[7] Tetreault LA, Karpova A, Fehlings MG. Predictors of outcome in patients with degenerative cervical spondylotic myelopathy undergoing surgical treatment: results of a systematic review. Eur Spine J. 2015;24 Suppl 2:236–251. doi: 10.1007/s00586-013-2658-z. 23386279

[8] Evans AR, Smith L, Bakhsheshian J, et al. Sarcopenia and the management of spinal disease in the elderly. Geroscience. 2025;47(2):1471–1484. doi: 10.1007/s11357-024-01300-2. 39138794 PMC11978579

[9] Moskven E, Bourassa-Moreau É, Charest-Morin R, et al. The impact of frailty and sarcopenia on postoperative outcomes in adult spine surgery: A systematic review of the literature. Spine J. 2018;18(12):2354–2369. doi: 10.1016/j.spinee.2018.07.008. 30053520

[10] Cruz-Jentoft AJ, Sayer AA. Sarcopenia. Lancet. 2019;393(10191):2636–2646. doi: 10.1016/S0140-6736(19)31138-9. 31171417

[11] Papadopoulou SK. Sarcopenia: a contemporary health problem among older adult populations. Nutrients. 2020;12(5):1293. doi: 10.3390/nu12051293. 32370051 PMC7282252

[12] Haase CB, Brodersen JB, Bülow J. Sarcopenia: early prevention or overdiagnosis. BMJ. 2022;376:e052592. doi: 10.1136/bmj-2019-052592. 35027442

[13] Cruz-Jentoft AJ, Bahat G, Bauer J, et al. Sarcopenia: revised European consensus on definition and diagnosis. Age Ageing. 2019;48(1):16–31. doi: 10.1093/ageing/afy169. 30312372 PMC6322506

[14] Beaudart C, McCloskey E, Bruyère O, et al. Sarcopenia in daily practice: assessment and management. BMC Geriatr. 2016;16(1):170. doi: 10.1186/s12877-016-0349-4. 27716195 PMC5052976

[15] Schönnagel L, Chiaparelli E, Camino-Willhuber G, et al. Spine-specific sarcopenia: distinguishing paraspinal muscle atrophy from generalized sarcopenia. Spine J. 2024;24(7):1211–1221. doi: 10.1016/j.spinee.2024.02.021. 38432297

[16] Albano D, Messina C, Vitale J, et al. Imaging of sarcopenia: old evidence and new insights. Eur Radiol. 2020;30(4):2199–2208. doi: 10.1007/s00330-019-06573-2. 31834509

[17] Tagliafico AS, Bignotti B, Torri L, et al. Sarcopenia: how to measure, when and why. Radiol Med. 2022;127(3):228–237. doi: 10.1007/s11547-022-01450-3. 35041137 PMC8960583

[18] Ozturk Y, Koca M, Burkuk S, et al. The role of muscle ultrasound to predict sarcopenia. Nutrition. 2022;101:111692. doi: 10.1016/j.nut.2022.111692. 35660496

[19] Chianca V, Albano D, Messina C, et al. Sarcopenia: imaging assessment and clinical application. Abdom Radiol (NY). 2022;47(9):3205–3216. doi: 10.1007/s00261-021-03294-3. 34687326 PMC8536908

[20] Hirase T, Haghshenas V, Bratescu R, et al. Sarcopenia predicts perioperative adverse events following complex revision surgery for the thoracolumbar spine. Spine J. 2021;21(6):1001–1009. doi: 10.1016/j.spinee.2021.02.001. 33561547

[21] Tsutsui S, Hashizume H, Iwasaki H, et al. Sarcopenia at the upper instrumented vertebra is more significantly associated with proximal junctional kyphosis after long fusion for adult spinal deformity surgery than osteopenia. J Clin Neurosci. 2023;116:13–19. doi: 10.1016/j.jocn.2023.08.012. 37597329

[22] Sun K, Zhu H, Huang B, et al. MRI-based central sarcopenia negatively impacts the therapeutic effectiveness of single-segment lumbar fusion surgery in the elderly. Sci Rep. 2024;14(1):5043. doi: 10.1038/s41598-024-55390-1. 38424180 PMC10904385

[23] Eleswarapu A, O’Connor D, Rowan FA, et al. Sarcopenia is an independent risk factor for proximal junctional disease following adult spinal deformity surgery. Global Spine J. 2022;12(1):102–109. doi: 10.1177/2192568220947050. 32865046 PMC8965302

[24] Hirabayashi K, Satomi K. Operative procedure and results of expansive open-door laminoplasty. Spine (Phila Pa 1976). 1988;13(7):870–876. doi: 10.1097/00007632-198807000-00032. 3143157

[25] Battaglia PJ, Maeda Y, Welk A, et al. Reliability of the Goutallier classification in quantifying muscle fatty degeneration in the lumbar multifidus using magnetic resonance imaging. J Manipulative Physiol Ther. 2014;37(3):190–197. doi: 10.1016/j.jmpt.2013.12.010. 24630770

[26] Pinter ZW, Wagner S, Fredericks D, Jr, et al. Cervical paraspinal muscle fatty degeneration is not associated with muscle cross-sectional area: qualitative assessment is preferable for cervical sarcopenia. Clin Orthop Relat Res. 2021;479(4):726–732. doi: 10.1097/CORR.0000000000001621. 33416225 PMC8083838

[27] Chiba K, Ogawa Y, Ishii K, et al. Long-term results of expansive open-door laminoplasty for cervical myelopathy – average 14-year follow-up study. Spine (Phila Pa 1976). 2006;31(26):2998–3005. doi: 10.1097/01.brs.0000250307.78987.6b. 17172996

[28] Caffard T, Arzani A, Verna B, et al. Association between cervical sagittal alignment and subaxial paraspinal muscle parameters. Spine (Phila Pa 1976). 2024;49(9):621–629. doi: 10.1097/BRS.0000000000004897. 38098290

[29] Koshimizu H, Sakai Y, Harada A, et al. The impact of sarcopenia on cervical spine sagittal alignment after cervical laminoplasty. Clin Spine Surg. 2018;31(7):E342–E346. doi: 10.1097/BSD.0000000000000657. 29863596

[30] Wilson JRF, Badhiwala JH, Moghaddamjou A, et al. Adverse effects of frailty on the outcomes of surgery for degenerative cervical myelopathy: results from a prospective multicenter international data set of 757 patients. J Neurosurg Spine. 2023;39(6):815–821. doi: 10.3171/2023.6.SPINE23461. 37728372

[31] Bokshan SL, Han AL, DePasse JM, et al. Effect of sarcopenia on postoperative morbidity and mortality after thoracolumbar spine surgery. Orthopedics. 2016;39(6):e1159–e1164. doi: 10.3928/01477447-20160811-02. 27536954

[32] Sang C, Chen X, Ren H, et al. Correlation between lumbar multifidus fat infiltration and lumbar postoperative infection: a retrospective case–control study. BMC Surg. 2020;20(1):35. doi: 10.1186/s12893-019-0655-9. 32093662 PMC7041265

[33] Zuckerman J, Ades M, Mullie L, et al. Psoas muscle area and length of stay in older adults undergoing cardiac operations. Ann Thorac Surg. 2017;103(5):1498–1504. doi: 10.1016/j.athoracsur.2016.09.005. 27863730

[34] Solla-Suarez P, Arif SG, Ahmad F, et al. Osteosarcopenia and mortality in older adults undergoing transcatheter aortic valve replacement. JAMA Cardiol. 2024;9(7):611–618. doi: 10.1001/jamacardio.2024.0911. 38748410 PMC11097099

[35] Martin L, Hopkins J, Malietzis G, et al. Assessment of computed tomography (CT)-defined muscle and adipose tissue features in relation to short-term outcomes after elective surgery for colorectal cancer: a multicenter approach. Ann Surg Oncol. 2018;25(9):2669–2680. doi: 10.1245/s10434-018-6652-x. 30006691

[36] Heus C, Smorenburg A, Stoker J, et al. Visceral obesity and muscle mass determined by CT scan and surgical outcome in patients with advanced ovarian cancer: a retrospective cohort study. Gynecol Oncol. 2021;160(1):187–192. doi: 10.1016/j.ygyno.2020.10.015. 33393479

[37] Addison O, Marcus RL, Lastayo PC, et al. Intermuscular fat: a review of the consequences and causes. Int J Endocrinol. 2014;2014:309570–309511. doi: 10.1155/2014/309570. 24527032 PMC3910392

[38] Liu S, Yang SD, Fan XW, et al. Analyses of effect factors associated with the postoperative dissatisfaction of patients undergoing open-door laminoplasty for cervical OPLL: a retrospective cohort study. J Orthop Surg Res. 2019;14(1):161. doi: 10.1186/s13018-019-1208-8. 31138291 PMC6540572

[39] Tamai K, Grisdela P Jr., Romanu J, et al. The impact of cervical spinal muscle degeneration on cervical sagittal balance and spinal degenerative disorders. Clin Spine Surg. 2019;32(4):E206–E213. doi: 10.1097/BSD.0000000000000789. 30762839

[40] Yoon SY, Moon HI, Lee SC, et al. Association between cervical lordotic curvature and cervical muscle cross-sectional area in patients with loss of cervical lordosis. Clin Anat. 2018;31(5):710–715. doi: 10.1002/ca.23074. 29575212

[41] Lee BJ, Park JH, Jeon SR, et al. Importance of the preoperative cross-sectional area of the semispinalis cervicis as a risk factor for loss of lordosis after laminoplasty in patients with cervical spondylotic myelopathy. Eur Spine J. 2018;27(11):2720–2728. doi: 10.1007/s00586-018-5726-6. 30105579

[42] Passias PG, Segreto FA, Horn SR, et al. Fatty infiltration of the cervical extensor musculature, cervical sagittal balance, and clinical outcomes: an analysis of operative adult cervical deformity patients. J Clin Neurosci. 2020;72:134–141. doi: 10.1016/j.jocn.2019.12.044. 31926664

[43] Passias PG, Segreto FA, Bortz CA, et al. Fatty infiltration of cervical spine extensor musculature: is there a relationship with cervical sagittal balance. Clin Spine Surg. 2018;31(10):428–434. doi: 10.1097/BSD.0000000000000742. 30371601

[44] Wang B, Qu R, Liu Z, et al. Comparison of postoperative pain and surgical outcomes between three types of modified muscle-sparing laminoplasty and conventional laminoplasty. Global Spine J. 2026. doi:10.1177/21925682241265625. PMC11571585 38910265

